# Ionocovalency and Applications 1. Ionocovalency Model and Orbital Hybrid Scales

**DOI:** 10.3390/ijms11114381

**Published:** 2010-11-03

**Authors:** Yonghe Zhang

**Affiliations:** American Huilin Institute, 13810 Franklin Ave, Queens, NY 11355, USA; E-Mail: y.zhang.huilin@gmail.com

**Keywords:** ionocovalency, molecular properties, electronegativity, theoretical chemistry

## Abstract

Ionocovalency (*IC*), a quantitative dual nature of the atom, is defined and correlated with quantum-mechanical potential to describe quantitatively the dual properties of the bond. Orbiotal hybrid *IC* model scale, *IC*, and *IC* electronegativity scale, *X_IC_*, are proposed, wherein the ionicity and the covalent radius are determined by spectroscopy. Being composed of the ionic function *I* and the covalent function *C*, the model describes quantitatively the dual properties of bond strengths, charge density and ionic potential. Based on the atomic electron configuration and the various quantum-mechanical built-up dual parameters, the model formed a *Dual Method* of the multiple-functional *prediction*, which has much more versatile and *exceptional* applications than traditional electronegativity scales and molecular properties. Hydrogen has *unconventional* values of *IC* and *X_IC_*, lower than that of boron. The *IC* model can agree fairly well with the data of bond properties and satisfactorily explain chemical observations of elements throughout the Periodic Table.

## 1. Introduction

In the valence-bond (VB) approach, the molecular wave function is written as a product of the state functions of the constituent atoms. This makes models based on the VB approximation intuitively appealing, as exemplified by the extreme usefulness of Lewis structures of “The Atom and the Molecule” [1] and by the wide acceptance of Pauling’s “Nature of the Chemical Bond” [2]. Before Lewis proposed his theory of the shared electron pair bond, bonding in some compounds could be satisfactorily explained on the basis of simple electrostatic forces between the positive and negative ions which are assumed to be the basic molecular units. The shared electron pair bond is known as the covalent bond, while the other type is the ionic bond.

Over the years, the description of the properties of the covalent and ionic bond remains largely qualitative and Pauling’s scale [3] due to not based on the electron configuration data, left a wide front for arguments and has lead to many different suggestions for the bond strengths [4–24].

In the present work, we defined and correlated ionocovalency (*IC*), a quantitative atomic dual nature of ionicity and *σ*- and spatial-covalency with the quantum-mechanical potential to describe quantitatively the dual properties of bonds. Orbital hybrid *IC* scale and *IC* electronegativity *X_IC_* scale are proposed wherein the ionicity and the covalent radius are determined by spectroscopy. Being composed of the ionic function *I* and the covalent function *C*, the model exhibits quantitatively the dual properties of bond strengths, charge density and ionic potential. Based on the atomic electron configuration, the quantum-mechanical built-up dual parameters and sub-models, which in turn exhibit various specific bond properties, the model formed a *Dual Method* of the multiple-functional *prediction* which has much more versatile and *exceptional* applications than traditional electronegativity scales and molecular properties. Hydrogen has its *unconventional* values lower than that of boron, residing on the borderline between the weak ionic and the weak covalent ions. The *IC* model can agree fairly well with the data of bond properties and satisfactorily explain chemical observations of elements throughout the Periodic Table.

## 2. Methodology

### 2.1. IC Model

Based on the VB approximation, the bond strengths can be considered mainly about the potential energy: the nuclear charge *Z* felt by valence electrons at the covalent boundary. And the term of Schrödinger’s Wave Equation incorporating bond strength is the potential energy *Ze*^2^*ψ*/*r.*

(2.1.1) $$-h^{2}\nabla^{2}\psi /8\pi^{2}m-Ze^{2}\psi /4\pi {ɛ}_{0}r=E\psi$$

Ionic bonds are omnidirectional. The nuclear charge *Z* possesses the power of ionizing radiation and radiates positive charge in all directions. Therefore, the nuclear charge *Z* is directly proportional to the bond strengths but has the delocalized *ionic* nature.

The covalent radius, *r_c_* is the other important part of the potential here. It is a distance from nucleus to a charge density wherein the bonding atoms are aligned and localized at very specific bond lengths, and it is inversely proportional to the bond strengths. In the case of the hydrogen atom, as Equation (2.1.1) shows, as the electron approaches the nucleus, the potential energy dives down toward minus-infinity, in order for the total energy *E* to remain constant, and its kinetic energy shoots up toward positive-infinity. So a compromise is reached in which theory tells us that the fall potential energy is just twice the kinetic energy, and the electron dances at an average distance that corresponds to the Bohr radius [25]. The calculation of the H_2_ molecule by Heitler and London showed that as the inter-nuclear distance decreased, the potential energy associated with interactions between nucleus and electrons dropped very markedly until a minimum was reached, and then (owing to the greater effect of internuclear repulsion at much smaller internuclear distances) to rise sharply [26]. The minimum corresponded fairly closely to the experimentally determined value of R, 0.74 Å (covalent radius = 0.37 Å, see below 4.2). Therefore, the covalent radius has the harmoniously localized *covalent* nature.

As atoms and molecules have the dual nature, ionocovalency, the bond strengths can be considered as a combination of the ionic and the covalent functions [10]. So the ionic function *I* can be considered as a function of the nuclear charge *Z: I*(*Z*); and the covalent function *C* can be considered as a function of the covalent radius *r_c_*: *C*(*r_c_*^−1^), wherein the reciprocal of *r_c_* can be defined as *atomic covalency.*

Ionocovalently, the bond strengths and electronegativity, therefore, can be accounted for on the basis of the dual nature of bonds, and functionally defined as ionocovalency, a product of the ionic and the covalent functions:

(2.1.2) $$IC=I(Z*)C(r_{c}^{-1})$$

And so the bond strengths, the potential energy and electronegativity have the *IC* framework of ionocovalency, which shows an effective ionocovalent potential, the attraction power that should be Pauling postulated.

According to the Bohr energy model

(2.1.3) $$E=-Z^{2}me^{4}/8n^{2}h^{2}{ɛ}_{0}^{2}=-RZ^{2}/n^{2}$$

we have derived the effective nuclear charge Z* from ionization energy and the effective principal quantum number n* [8,9]:

(2.1.4) $$Z*=n*{\left(I_{z}/R\right)}^{½}$$

where *I_z_* is the ultimate IE. *R* is the Rydberg constant, *R =* 2π^2^*μ*^4^2*e*^4^/*h*^2^ *=* 13.6 eV, *h* is Planck’s constant. Substituting Equation (2.1.4) into (2.1.2), we can naturally correlate the bond properties to the quantum-mechanics, and get the *IC model*:

(2.1.5) $$I(I_{z})C(n*r_{c}^{-1})=n*{\left(I_{av}/R\right)}^{½}r_{c}^{-1}$$

where the effective principal quantum number n* is related to the electron energy, distribution and the distance from the nucleus. Hence, n* can be considered as an energy of ionic function and also as a spatial distance of covalent function. The n*r_c_^−1^, which is related to the spatial overlap, can be defined as *spatial-covalency* of covalent function (*r_c_*^−1^ is a *linear- or σ-covalency*).

### 2.2. IC Electronegativity X_IC_

As an application of ionocovalency, by Plotting Pauling values of *X_p_* against n*(I_av_/*R)*^½^*r_c_*^−1^, we obtain a new *IC-potential* electronegativity

(2.1.6) $$X_{IC}=0.412n*{\left(I_{av}/R\right)}^{½}r_{c}^{-1}+0.387$$

Based on the above *IC* model, our previous electronegativity scale *X_z_* [8,9] can be accounted for *IC-force*:

(2.1.7) $$IC=I(V)C(r^{-1})=I(Z*)C(r^{-2})=n*{\left(I_{z}/R\right)}^{½}r_{c}^{-2}$$

(2.1.8) $$X_{z}=0.241n*{\left(I_{z}/R\right)}^{½}r_{c}^{-2}+0.775$$

In Equation 2.1.5 and 2.1.6, *I_av_* is *ionicity* (the average *IE* of valence shell electrons) determined by the following *IC orbital hybrid bonding procedures*. The diagrams are adapted from those in the excellent article by Blaber [27].

### 2.3. IC Orbital Hybrid Bonding Procedures

(1) **Ionization promotion**: As a valence shell fills, the successive increased ionization energy of an electron would provide the promotion energy for hybrid orbital formation e.g., consider gaseous molecules of BeF_2_. The fluorine atom has the electron configuration: 1*s*^2^2*s*^2^2*p*^5^. The beryllium atom has the electron configuration: 1*s*^2^2*s*^2^.

**Figure:**
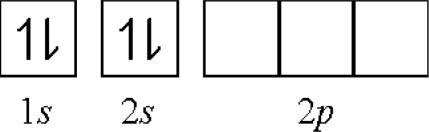


In the ground state, there are no unpaired electrons. However, the beryllium atom could obtain an unpaired electron by promoting an electron from the 2*s* orbital to the 2*p* orbital by its lower first *IE* (9.32 eV). The beryllium atom can now forms two covalent bonds with fluorine atoms:

**Figure:**
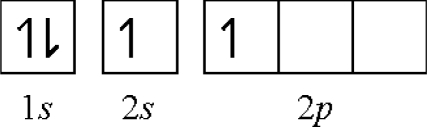


(2) **Ionicity hybridization**: We can combine functions for the 2*s* and 2*p* electrons to produce a “hybrid” orbital for both electrons. The charge transfer and charge distribution would occur from the higher energy level of the 2*p* orbital to the lower energy level of the 2*s* orbital to form an energy-lower and identical 2*sp* hybrid orbital. The ideas developed are adequate for calculation of the charge density identically distributed. The *IEs* of the 2*s* and 2*p* electrons can be averaged to result in a hybridizing *ionicity*, *I_av_*:

(2.1.9) $$I_{av}=n^{-1}\Sigma_{i=1}^{n}I_{i}$$

where *I_i_* is the IE of single electron of valence shell, *n* is number of valence shell electrons.

By Equation (2.1.9), we get an average IE: *I_av_* = 13.76 eV from the first IE (9.32 eV) and second IE (18.2 eV):

first: Be[He](2*s*)^2^ → Be*^+^*[He] (2*s*)^1^ − 9.32 eV
second: Be*^+^*[He](2*s*)^1^ → Be^2+^[He] − 18.20 eV

of 2*s* electrons for 2*sp* electrons:

**Figure:**
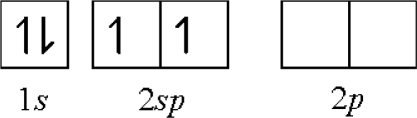


(3) **Ionocovalency**: by localizing the hybridized ionicity at the covalent boundary *r_c_* to form an ionocovalent bond of 2*sp* hybrid orbitals:

**Figure:**
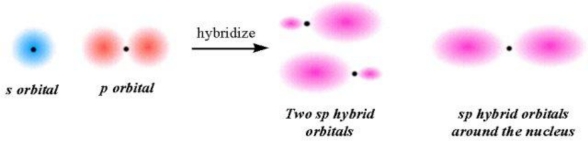


Hence, for beryllium, *IC = I*(*I_av_*)*C*(n*r_c_^−1^) = n*(*I_av_*/*R*)^½^*r_c_*^−1^ = 1.99(13.76/13.6)^½^ (0.970)^−1^ = 2.064 And *X_IC_* *=* 0.412 n*(*I_av_*/*R*)^½^ *r_c_*^−1^ *+* 0.387 *=* 0.412*1.99(13.76/13.6)^½^(0.970) ^−1^ + 0.387 = 1.237.

Similarly, an *s* orbital can also mix with all three *p* orbitals in the same subshell. For CH_4_ by Equation (2.1.9), we get ionicity: *I_av_* = 37.015 eV from the first *IE* (11.26 eV), second *IE* (24.40 eV), third *IE* (47.90 eV) and fourth *IE* (64.50 eV)

- first: C[He](2*s*)^2^(2*p*)^2^ → C*^+^*[He](2*s*)^2^(2*p*)^1^ − 11.26 eV
- second: C*^+^*[He](2*s*)^2^(2*p*)^1^ → C^2+^[He](2*s*)^2^ − 24.40 eV
- third: C^2+^[He](2*s*)^2^ → C^3+^[He](2*s*)^1^ − 47.90 eV
- fourth: C^3+^[He](2*s*)^1^ → C^4+^[He] − 64.50 eV

of the all 2*s* and 2*p* electrons and form 2*sp^3^* hybrid orbitals:

**Figure:**
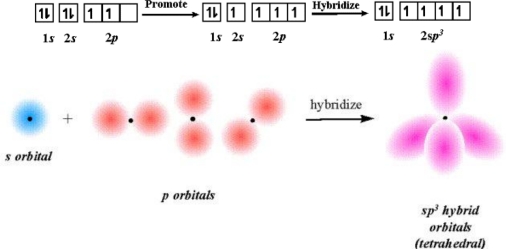


The *IE* is taken from Mackay *et al.* [28], the covalent radius *r_c_*, is taken from Pauling [29], Batsanov [30], Cordero *et al.* [31], Rappe *et al.* [32] and the effective principal quantum number n* is from our previous work [8,9]. The ionocovalency scale, *IC*, calculated from Equation (2.1.5) is listed in Chart 1 and the electronegativity scale, *X_IC_* calculated from Equation (2.1.6) are listed in Table 1.

Based on the *IC* model, ionocovalency can be finally defined as “*the effective potential caused by the ionicity on a bonding pair of electrons at the localized covalent boundary in different valence hybrid orbital states, forming an ionocovalent charge density*”.

## 3. Results

### 3.1. General Trend of Periodic Table

**Natural Values:** Chart 1 shows that *IC* has a same value range as that of its atomic core charge. This phenomenon is particularly noticeable for the top period elements which have not yet experienced much shielding effects. The atomic core charge is an effective nuclear charge and is always markedly less than the actual nuclear charge *Z*, but, as the shielding is not perfect, the core charge increases as *Z* increases, but more slowly. In the *IC* model, the effective nuclear charge is determined by natural *IE*, not from the calculation by shielding constants.

**Ionocovalent Continuum:** Chart 1 and Table 1 show that the ionocovalency *IC* and the electronegativity *X_IC_* exhibit evidently the ionocovalency character. The greater the *IC* and the *X_IC_*, the more covalent and the less ionic the cation is, and *vice versa*. Generally, across the period, the more right-hand-side an element is, the more covalent it is. And the more down-ward an element is, the more ionic it is. The *IC* and *X_IC_* increase from the lower left to the upper right of the Periodic Table across the *s* and *p* blocks, and decrease down most columns. Trends parallel periodic trends in *IE*.

The ionocovalency is a continuum which is certainly an improvement over the old ionic-*versus*-covalent dichotomy. In so-called ‘pure’ ionic bonding, an electron is transferred completely from one atom to another, once this transfer is complete, the *IC* potential will act to try to pull this electron back to its parent ion. If *IC* partially succeeds then there will be some electron density, n*(I_av_/*R)*^½^*r_c_*^−1^, in the region in between the ions, which is the situation in a covalent bond. Thus ‘pure’ ionic and ‘pure’ covalent bonds could be seen as two extremes of an *IC continuum*. And so *a covalent scale has the ionic degree.*

**Energy-lowered Hybrid Bonding:** The ionocovalent bonding procedures runs a charge promotion, charge distribution and energy-lowered hybrid sequence. Using Equation 2.1.5. and 2.1.6., we get the energy-lowered hybrid *IC* and *X_IC_* values which are evidently lower than that of unhybridized values. For example, for carbon we have the energy-lowered 2*sp^3^* hybrid *IC* = 4.320 and *X_IC_* = 2.167. In the unhybridized situation, *IC* would be as higher as 5.702 and all unhybridized conventional electronegativities are as higher as than 2.5 for carbon.

### 3.2. Hydrogen

Pauling’s scale is estimated from the bond dissociation energies of two atoms, hydrogen and chlorine, and then arbitrarily extended to all elements not based on the quantitative configuration energy data [3].

**Figure 1.**
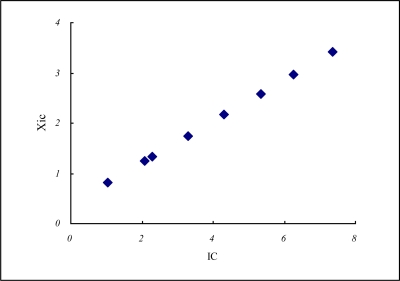
*IC vs. X_IC_* for hydrogen and the top elements indicated in Table 2.

Hydrogen has the lowest energy, *E* = −13.6 eV. Batsanov has an experimental covalent radius of 0.37 Å [30] equal to the Heitler-London’s half H-H value *R* (*R* = 0.74 Å) [26]. Based on these spectroscopic data and the *IC* model (Equation 2.1.5), we reach its *IC* value of 2.297 eV and its electronegativity, *X_IC_* of 1.333 (Table 2 and Figure 1) that is not that conventionally high as 2.2.

The result is strongly supported by the point-charge distribution in hydrides (Table 3) proposed by Mo [33], which shows that in the typical ionic LiH (lithium monohydride) hydrogen gains point charge of 0.783, in the weak ionic BeH (beryllium monohydride) gains only 0.044, but in the covalent BH (boron monohydride) starts to loss point charge. That means that the ionocovalency and the electronegativity of hydrogen are smaller than that of boron. And moreover, the data of electric dipole moments for AlH (aluminum monohydride) (Table 4), proposed by the National Institute of Standards and Technology [34], shows that the aluminum end of the dipole is negative. That means that the ionocovalency and the electronegativity of hydrogen are smaller than that of aluminum.

Hydrogen has one valency orbital and a single electron. It can be an anion to form an ionic bond by gaining another electron and it can be a cation to form a covalent bond by sharing another electron. Therefore, the *IC* and the *X_IC_* values of hydrogen happen to lie on the border between the weaker ionic beryllium and the weaker covalent boron (Chart 1 and Table 1). We can assign the *IC* value of hydrogen (2.297) as a standard to estimate the ionocovalent character of the cations. The cations with *IC* values smaller than that of hydrogen we call the ionic cations and those with *IC* values greater than that of hydrogen we call the covalent cations. The cations with *IC* values greater than that of beryllium (2.064) and smaller than that of boron (3.291) we might call borderline cations. The greater the *IC* than that of hydrogen, the more covalent and the less ionic the caton is, and *vice versa*.

### 3.3. Diagonal Relationship (Top Periods)

Chart 2 and Chart 3 show that the *IC* and the *X_IC_* scales of the top period rationalize an interesting empirical observation of a similar situation that exists for the pairs of elements. The first element in a given family of the periodic chart tends to resemble the second element in the family to the right as indicated below:

**Chart 1.** Ionocovalency.

**H**																	**He**
(1)2.297																	(2)5.173
**Li**	**Be**											**B**	**C**	**N**	**O**	**F**	**Ne**
(1)1.023	(2)2.064											(3)3.291	(4)4.302	(5)5.329	(6)6.273	(7)7.370	(4)6.949
													(2)2.998	(3)4.030	(2)3.648		(2)5.203
Na	Mg											**Al**	**Si**	**P**	**S**	**Cl**	**Ar**
1.130	(2)1.933											(3)2.730	(4)3.371	(5)4.355	(6)5.165	(7)6.048	(4)4.845
												(1)1.585	(2)2.322	(4)3.872	(4)4.093	(5)4.977	(2)3.762
														(3)3.286	(2)2.121	(1)2.851	
**K**	**Ca**	**Sc**	**Ti**	**V**	**Cr**	**Mn**	**Fe**	**Co**	**Ni**	**Cu**	**Zn**	**Ga**	**Ge**	**As**	**Se**	**Br**	**Kr**
(1)0.999	(2)1.617	(3)2.494	(4)3.374	(5)4.359	(6)5.287	(7)6.012	(6)5.520	(4)4.261	(4)4.537	(3)3.918	(2)2.772	(3)3.281	(4)3.895	(5)4.502	(6)5.209	(7)5.941	(6)5.422
			(3)2.824	(4)3.772	(5)4.691	(6)5.426	(5)4.879	(3)3.591	(3)3.802	(2)3.155	(1)2.331	(1)1.841	(2)2.639	(3)3.364	(4)4.090	(5)4.869	(4)4.562
			(2)2.258	(3)3.143	(4)4.058	(5)4.810	(4)4.203	(2)2.874	(2)3.029	(1)2.343				(1)2.422	(2)3.146	(1)2.815	(2)3.538
				(2)2.502	(3)3.395	(4)4.161	(3)3.431	(1)2.113	(1)2.330								
					(1)2.077	(2)2.719	(1)2.253										
						(1)2.185											
**Rb**	**Sr**	**Y**	**Zr**	**Nb**	**Mo**	**Tc**	**Ru**	**Rh**	**Pd**	**Ag**	**Cd**	**In**	**Sn**	**Sb**	**Te**	**I**	**Xe**
(1)0.988	(2)1.576	(3)2.332	(4)3.716	(5)4.043	(6)4.964	(7)5.593	(8)6.394	(6)5.450	(5)4.842	(3)3.592	(2)2.658	(3)2.923	(4)3.601	(5)4.057	(6)4.641	(7)5.339	(8)5.553
			(3)2.267	(4)3.575	(5)4.547	(6)5.090	(7)5.890	(4)4.233	(4)4.277	(2)2.973	(1)2.174	(1)1.678	(2)2.472	(3)3.036	(4)3.675	(5)3.817	(6)4.667
			(2)1.884	(3)3.052	(4)3.980	(5)4.563	(6)5.355	(3)3.638	(3)3.672	(1)2.147					(2)2.832	(1)2.530	(4)3.900
				(2)2.532	(3)3.318	(4)4.008	(5)4.791	(2)2.993	(2)3.042								(2)3.044
				(1)2.040	(2)2.760	(3)3.425	(4)4.189	(1)2.287									
					(1)2.155	(2)2.762	(3)3.525										
		Δ				(1)2.218	(2)2.924										
**Cs**	**Ba**	**La**	**Hf**	**Ta**	**W**	**Re**	**Os**	**Ir**	**Pt**	**Au**	**Hg**	**Tl**	**Pb**	**Bi**	**Po**	**At**	**Rn**
(1)0.992	(2)1.646	(3)2.185	(4)3.624	(5)4.393	(6)5.178	(7)5.874	(8)6.616	(6)5.683	(6)5.640	(5)5.033	(2)3.118	(3)3.307	(4)3.780	(5)4.273	(6)4.707	(7)5.430	(8)5.567
			(3)3.170	(4)3.924	(5)4.697	(6)5.383	(7)6.122	(5)5.107	(5)5.056	(3)3.965	(1)2.653	(1)1.887	(2)2.574	(3)3.162	(4)3.700	(5)4.419	(6)5.046
			(2)2.691	(3)3.462	(4)4.200	(5)4.881	(6)5.608	(4)4.478	(4)4.518	(2)3.412					(3)3.285	(3)3.522	(4)4.183
				(2)3.056	(3)3.707	(4)4.344	(5)5.066	(3)3.910	(3)3.963	(1)2.689					(2)2.811	(1)2.473	
				(1)2.473	(2)3.261	(3)3.795	(4)4.483	(2)3.324	(2)3.405								
						(2)3.237	(3)3.869	(1)2.831	(1)2.749								
						(1)2.597	(2)3.370										
		ΔΔ					(1)2.779										
**Fr**	**Ra**	**Ac**	Δ	**Ce**	**Pr**	**Nd**	**Pm**	**Sm**	**Eu**	**Gd**	**Tb**	**Dy**	**Ho**	**Er**	**Tm**	**Yb**	**Lu**
(1)0.959	(2)1.400	(3)2.093		(4)3.074	(4)3.037	(3)2.572	(3)2.606	(3)2.604	(3)2.385	(3)2.637	(4)3.301	(3)2.730	(3)2.754	(3)2.780	(3)2.829	(3)2.649	(3)2.592
				(3)2.506	(3)2.368	(2)2.050			(2)1.858	(2)2.213	(3)2.686				(2)2.286	(2)2.112	
			ΔΔ	**Th**	**Pa**	**U**	**Np**	**Pu**	**Am**	**Cm**	**Bk**	**Cf**	**Es**	**Fm**	**Md**	**No**	**Lw**
				(4)2.789	(3)2.279	(3)2.316	(4)2.341	(4)2.354	(4)2.349	(3)2.165	(3)2.215	(3)2.229	(3)2.262	(3)2.278	(3)2.309	(3)2.323	(3)3.900
				(3)2.431													

Note: Some ions which might not actually exist are included here just for research reference.

**Chart 2.** *IC*-diagonal relationship.

Be^2+^ (2.252)	B^3+^ (3.291)	C^4+^ (4.320)	N^5+^ (5.554)	O^6+^ (6.939)
Mg^2+^ (1.933)	Al^3+^ (2.730)	Si^4+^ (3.371)	P^5+^ (4.355)	S^6+^ (5.165)

**Chart 3.** *X_IC_*-diagonal relationship.

Be^2+^ (1.315)	B^3+^ (1.743)	C^4+^ (2.167)	N^5+^ (2.675)	O^6+^ (3.246)
Mg^2+^ (1.184)	Al^3+^ (1.418)	Si^4+^ (1.776)	P^5+^ (2.181)	S^6+^ (2.515)

**Chart 4.** *I_av_*-diagonal relationship.

Be^2+^ (13.76)	B^3+^ (23.800)	C^4+^ (37.015)	N^5+^ (53.406)	O^6+^ (72.020)
Mg^2+^ (11.325)	Al^3+^ (17.763)	Si^4+^ (25.763)	P^5+^ (35.358)	S^6+^ (46.077)

**Chart 5.** n*/*r_c_*-diagonal telationship.

Be^2+^ (2.238)	B^3+^ (2.488)	C^4+^ (2.618)	N^5+^ (2.803)	O^6+^ (3.015)
Mg^2+^ (2.119)	Al^3+^ (2.189)	Si^4+^ (2.449)	P^5+^ (2.701)	S^6+^ (2.806)

The reason for this relationship is that the pairs of element have approximately similar ionocovalency, *IC = I*(*I_av_*)*C*(n*r_c_^−1^), due to the approximately similar ionic function *I*(*I_av_*) (Chart 4) and covalent function *C*(n*r_c_^−1^) (Chart 5). Because of the electron configuration nature of the elements, the downwards vertical trend in decreasing covalency *r_c_*^−1^ is the opposite of the downwards vertical trend in increasing principal quantum number n*, and in this section of the Periodic Table the two opposing trends in *C*(n*r_c_^−1^) approximately cancel each other, resulting in similar values of ionicity, *I*(*I_av_*).

### 3.4. Carbon, Sulfur, P-elements and Hydrogen

There are some arguments about the values of electronegativities of carbon, sulfur, selenium, tellurium, iodine and hydrogen [22]. Chart 1 shows *IC* value*s* in the order:

$$Se^{2+}(3.146)>S^{2+}(3.121)>C^{2+}(2.998)>Te^{2+}(2.832)>I^{+}(2.530)>H^{+}(2.297)$$

The results are consistent with the observations that hydrides H_2_Se, H_2_S, H_2_C, H_2_Te and HI form H_3_O*^+^* ions in water [35].

As Thomas reviewed, the electronegativity of carbon and sulfur in most of the scale are almost identical. The key point, however, so far as their role as poisons is concerned, is that they differ markedly in the distance at which they sit on the nickel overlayers [36]. The calculations for these locations show that sulfur is very much stronger than carbon as a poison.

The results are also consistent with the experiment data of the dipole moment, which indicates that the electron clouds on the C-S and C-I bond in the molecules CS_2_ and CI_4_ are close to the sulfur end and the iodine end, respectively [37]. From *IC* model data (Chart 4), we can see that *S*^6^*^+^* has a greater ionicity than that of *C*^4^*^+^*: I_av_ (*S*^6^*^+^* = 46.077, *C*^4^*^+^* = 37.015), although they have the close spatial covalency, n*r_c_^−1^ (*C*^4^*^+^* = 2.618, *S*^6^*^+^* = 2.805) (Chart 5).

### 3.5. 3d and 4f Electron Inefficient Screening (p-Block)

As Chart 1 and Table 1 show, the *IC* and *X_IC_* run the same uneven trend that is expected to decrease down a group with the features in terms of As^3+^(*IC* = 3.364, *X_IC_* = 1.773) and Bi^3+^(*IC* = 3.162, *X_IC_* = 1.690) both having higher than expected values, comparing those of P^3+^(*IC* = 3.286, *X_IC_* = 1.741) and Sb^3+^(*IC* = 3.036, *X_IC_* = 1.638). This apparent anomaly derives from the filling of the 3*d* row prior to gallium and the *4f* row prior to thallium, both of which lead to higher effective nuclear charges (Table 1) than the previous periods as a result of inefficient screening of the nuclear charge by the 3*d* and 4*f* electrons, respectively.

### 3.6. Standard Potential Redox E^0^ (Transition Elements)

The IC values with a trend of decreasing at *d*^5^ and *d*^10^ agree well with the variation in values of *E*^0^ *(M*^2^*^+^*/*M)* [38] as a function of *d*^n^ configuration for the first row of transition metals; the *d*^0^ corresponds to M = Ca (Table 5).

### 3.7. Inert Pair Effect (6s^2^ Elements)

The *IC* model, based on the VB approximation’s intuitive appeal and determined by covalent radius and ionization energy, is in accord with the relativistic effects with which contributions to the unusual chemistry of the heavier elements are two principal consequences. First, the *s* orbitals become more stable. Second, *d* and *f* orbitals expand and their energies are less.

For the inert pair effect in Tl(I), Pb(II), and Bi(III), the relativistic effects can give a qualitative verbalization: “The s orbitals of the heavier elements become more stable than otherwise expected” [39]. In the *IC* model, as Table 6 shows, the effect is attributable to the fact that the bond property in this case is controlled by the ionic function *I*(*I_z_*, *I_av_*). They are more stable in ionic compounds than in the entirely covalent form. Their IEs for forming higher covalent bonds are too much higher to form a stable hybridizing ionicity *I_av_*:

### 3.8. Color of Copper, Silver and Gold

According to the relativistic effects: “The *s* orbitals of the heavier elements become more stable than otherwise expected” [39], we can only give a qualitative overview on the color of gold and silver, but this has nothing to do with the color of copper.

In the *IC* model, the phenomenon of “the Color of Copper, Silver and Gold” is attributable to the fact that their bond structure is controlled by their ionocovalency dual properties and we can get a satisfactory explanation by the Dual method.

As Table 7 shows, with the increase of the contraction of the *s* orbital, the outer *d* orbitals expand and their ionicities *I_av_* are decreased from Cu^3+^ via Ag^3+^ to Au^3+^, however, with the increase of their effective principle quantum number n* the covalency *r_c_*^−1^ decreases from Cu^3+^ to Ag^3+^ but increases from Ag^3+^ to Au^3+^, which causes the spatial covalency n*r_c_^−1^ of Cu^3+^ higher than that of Ag^3+^ but close to Au^3+^, leading to same trend in ionocovalency.

This quantitative trend in their structure and energy nicely reflect their character of color. Copper and gold are the only two elemental metals with a natural color other than gray or silver, which depends on their ionic delocalizing property of “electron sea” that is capable of absorbing and re-emitting photons over a wide range of frequencies. Copper in its liquefied state, a pure copper surface without ambient light, appears somewhat greenish, a characteristic shared with gold.

## 4. Applications

### 4.1. Covalency Result Is Retrieved

Villesuzanne *et al.* proposed the study: “New considerations on the role of covalency in ferroelectric niobates and tantalites” [23]. Here, covalency means the amount of mixing of oxygen 2*p* and metal *d* orbitals to form valence bands; it is evaluated quantitatively through the computation of the crystal orbital overlap population (COOP). The energies of Ta 5*d* and Nb 4*d* atomic orbitals are the same in EHTB parameters. The bond lengths are equal too, as found experimentally. The difference in COOP’s occurs because of larger radial extension of Ta 5*d* compared to Nb 4*d* orbitals, leading to a greater overlap with oxygen 2*p* orbitals. The fact that Ta^5+^-O bonds are more covalent than Nb^5+^-O bonds is due to a larger radial expansion of Ta *5d* orbitals. This effect is not accounted for in Pauling electronegativity scales [3], which give information on the energy difference between valence orbitals, not on their spatial overlap. The arguments led to the opposite assumption of reference [24] concerning the covalency of Ta^5+^-O and Nb^5+^-O bonds from Pauling electronegativity X_p_: Ta(1.5) < Nb(1.6).

In their later paper, they proposed that the explicit calculation of the electronic structure—COOP’s in particular—gives a larger covalency for Ta^5+^-O bonds than for Nb^5+^-O bonds. This result is retrieved in the Allred and Rochow scale [7] and in Zhang electronegativity scales for ions [9]. The results can be fairly well accounted in *IC* model: the energies of Ta 5*d* and Nb 4*d* atomic orbitals are the same in EHTB parameters due to having similar atomic ionicity *I_av_* of 24.89 and 27.02, respectively. The bond lengths are equal due to having similar linear covalency *r_c_*^−1^ of 0.745 and 0.745, respectively. The big difference is the spatial covalency, n*r_c_^−1^, in *I*(*I_av_*)*C*(n*r_c_^−1^) = n*(*I_av_*/*R*)^½^*r_c_*^−1^. The Ta 5*d* orbitals, compared to Nb 4*d* orbitals, involve greater spatial covalency, n*r_c_^−1^, (Ta^5+^ = 3.246, Nb^5+^ = 2.869), leading to a greater overlap with oxygen 2*p* orbitals and a greater *IC*: Ta^5+^ (4.393) > Nb^5+^ (4.043) and *X_IC_*: Ta^5+^(2.197) > Nb^5+^(2.053).

### 4.2. Mössbauer Parameters δ and Δ

**Figure 2.**
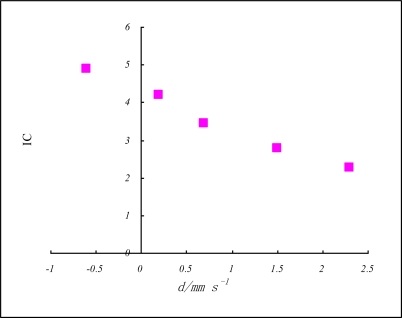
*IC vs*. *δ* for iron-57 as indicated in Table 9.

As the *IC* model, n*(I_av_/*R)*^½^*r_c_*^−1^, is defined as ionocovalent density of the effective nuclear charges at covalent boundary, it is strongly related with the Mössbauer parameters δ and *Δ* [40,41]. The value of the isomer shift, δ, depends particularly on the density of *s* electrons at the nucleus. Therefore, in *iron-57* an increase in electron density causes a negative isomer shift; since *d* electrons tend to shield the nucleus slightly from the *s* electrons, the value of δ falls as the number of *d* electrons in the iron atom falls. Mean values of δ [42], Z* and *IC* for some oxidation states of iron are shown in Table 8 and Figure 2.

### 4.3. Effective Polarizing Power and Fajans Rules

Fajans suggested the rules to estimate the extent to which a cation could polarize an anion and thus induce covalent character. This Fajans phenomenon happens to be the *IC*-potential, the ionocovalency, the effective ionic potential (or the effective polarizing power), n*(*I_av_*/*R*)^½^*r_c_*^−1^.

The simple form of the ionic potential considered the valence charge of the ion with respect to its size. The valence charge is numerically equal to the number of valence electrons of the ion. In some cases we may consider the effective nuclear charge Z*. For two ions of the same actual nuclear charge, Hg^2+^ and Ca^2+^, the Hg^2+^ has the higher effective nuclear charge Z* (4.490) and the *IC* (3.118), it is considerably more polarizing and its compounds are considerably more covalent than those of Ca^2+^ which has the smaller effective nuclear charge Z* (2.807) and the *IC* (1.617). So we have their related melting points HgCl_2_ = 276 and CaCl**_2_** = 772. Comparisons of more compounds are listed in Table 9 (below).

### 4.4. Melting Points and Bond Properties

Table 9 shows that for the covalent bonding, the increased covalent bonding resulting from increasing the ionicity *I_av_*, the *σ^−^* covalency *r_c_*^−1^ or the spatial covalency n*r_c_^−1^ can lower the transition temperatures. The melting points decrease with increasing the covalency *r_c_*^−1^ and the spatial covalency n*r_c_^−1^.

However, for ionic bonding (see 4.5.), the ionic compounds are characterized by very strong *IC* potentials holding the ions together. Increasing the ionic function *I*(Z*, *I_av_*) tends to increase the lattice energy of a crystal. For compounds which are predominantly ionic, increased ionic function *I*(Z*, *I_av_*) or covalent function *C*(*r_c_*^−1^, n*r_c_^−1^) will result in increased melting points.

### 4.5. Lattice Energy

The *IC* model Equation 2.1.5 is correlated with the electrostatic energy of a cation in Born-Landé equation of the lattice energy

(4.5.1) $$U=-Z^{2}e^{2}AN/4\pi {ɛ}_{0}r(1-n^{-1})$$

The both equations reveal how ionic bond strengths vary with the cation ionic charges and inversely with the distance between ions in the lattice. The IC gives a reasonable correlation to the lattice energy as shown in Table 10 and Table 11.

### 4.6. Lowis Acid Strengths

As we have described [8–10], the stability of a metal complex (the strength of metal-ligand bond) should be a function of the electron-attraction power of the metal. The *IC* value agrees fairly well with the lattice energy and the crystal field stabilization energy (*CFES*):

**Table 10.** Parameters and lattice energies, −U.

**Compound**	**Cation**	Z*	***I_av_***	**r_c_^−1^**	n*r_c_^−1^	***X_IC_***	***IC***	***U***(**kJmol^1^**) [44]
LiH	Li^+^	1.253	5.39	0.816	2.238	0.808	1.023	905.4
NaH	Na^+^	1.777	5.14	0.636	1.838	0.853	1.13	810.9
KH	K^+^	1.949	4.34	0.513	1.769	0.799	0.999	714.2

AgF	Ag^+^	2.874	7.58	0.747	2.875	1.271	2.147	954
NaF	Na^+^	1.777	5.14	0.636	1.838	0.853	1.13	903.9
KF	K^+^	1.949	4.34	0.513	1.769	0.799	0.999	801.2

AgCl	Ag^+^	2.874	7.58	0.747	2.875	1.271	2.147	904
TlCl	Tl^+^	2.922	6.11	0.646	2.815	1.164	1.887	732
KCl	K^+^	1.949	4.34	0.513	1.769	0.799	0.999	697.9
RbCl	Rb^+^	2.134	4.18	0.455	1.75	0.787	0.97	677.8

Table 11 shows that the *IC* values correlate with the lattice energies derived from Born-Haber cycle data for MCl_2_ where M is a first row *d*-block metal; the point for *d*^0^ corresponds to CaCl_2_. (Data are not available for scandium where the stable oxidation state is +3) [38].

Crystal field stabilization energy (*CFES*) causes to *d* level an effect upon thermodynamic stability of complex ions. Owing to the overall contraction in size on traversing a period from left to right, there is an increase in *CFES* from Ca^2+^ to Zn^2+^ and same trend occurred in *IC* and *X_IC_*, For weak-field ligands (Table 12), the formation constants log *β* [38] follow the uneven trend and the *IC* and *X_IC_* run in same way: Mn^2+^ < Fe^2+^< Co^2+^ < Ni^2+^ < Cu^2+^ < Zn^2+^.

## 5. Dual Method

We couldn’t expect “*verbum sat sapienti*”, but we have a dual parameter group that can dialectically serve many purposes. When *IC* and *X_IC_* correlate with some properties, their dual component parameters and sub-models would give some reason to an observation or find some clue to a new idea. See above all parameters, sub-models and observations to which we have applied the Dual Method. Further examples are as follows:

### 5.1. An Interesting Comparison

In Table 13 an interpretation for an interesting comparison can be made between the predominantly ionic species CsF and BaF_2_ and the more covalent species KBr and CaBr_2_. For ionic species, the bond strengths are controlled by the ionic function *I*(*I_av_*). Doubling the *I_av_* from 3.890 to 7.605 in the highly ionic fluorides produces the expected increase in lattice energy and correspondingly doubles the transition temperatures (from 684 °C to 1,28 °C). For covalent bonding, the covalent function *C*(n*r_c_^−1^) is controlling factor. The little change in covalency *r_c_*^−1^: 0.513, 0.576 and spatial covalency n*r_c_^−1^: 1.769, 1.987 produces the expected little change in transition temperatures (from 730 °C to 765 °C), despite the doubling of *I_av_* from 4.340 to 9.345.

### 5.2. “Inverted” Sodium-Lithium Electronegativity

Table 14 shows why electronegativity of sodium is higher than that of lithium. When we correlate *IC* or *X_IC_* with lattice energies, there is the “inverted” sodium-lithium electronegativity [8,9,45]; that Li*^+^* has unexpectedly low values of *IC* and *X_IC_*. However, we can ask *I*(*I_av_*, Z*) and *C*(n*, *r_c_*^−1^) to dialectically explain it: after 1st filling of *p* orbitals, Na*^+^* reaches a much higher effective nuclear charge Z*(1.777) than that of Li*^+^*(1.253). The spatial covalency n*r_c_^−1^(Na*^+^* = 1.838, Li*^+^* = 1.624) does not cancel the higher effective nuclear charge Z* anymore. Dialectically, however, Li*^+^* still has higher ionicity, *I_av_*(5.390) and covalency *r_c_*^−1^(0.816) than that of Na*^+^*(*I_av_* = 5.140, *r_c_*^−1^ = 0.636) although covalency is not so important in lattice energy.

### 5.3. Predicting Raw Material for InN Nanocrystals

Changzheng *et al.* [46] presented an effective synthetic protocol to produce high quality InN nanocrystals using indium iodide (InI_3_). There has been a question: “Is it possible for high-quality InN to be synthesized from indium halides?” The positive answer has been found in their work using InI_3_. Concerning the four kinds of indium halides, InF_3_, InCl_3_, InBr_3_, and InI_3_, InI_3_ has the strongest covalent ability. As is known, when two atoms form a chemical bond, the greater the difference between the electronegativity values for the two atoms, the more ionic the chemical bond between them [8–10].

According to the *IC* model, in the effective polarizing power, n*(*I_av_*/*R*)^½^*r_c_*^−1^, both the effective principle quantum number, n*, and the covalent radius, *r_c_*, for halogens are increased in the order: F < Cl < Br < I (Table 1). The polarizability of the anion will be related to its “softness”; that is, to the deformability of its electron cloud. Both increasing n* and *r_c_* will cause this cloud to be less under the influence of the nuclear charge of the anion and more easily influenced by the charge on the cation. So concerning the four kinds of indium halides, InI_3_ is more covalent than the other three. And it is possible for high-quality InN to be synthesized from indium iodide (InI_3_).

Comparison of melting points for the anion pairs KF/KBr and CaCl_2_/CaBr_2_ from Table 9 can be treated in same way. KBr and CaBr_2_ are more covalent and have lower melting points than KF and CaCl_2_ respectively (see 4.4.).

## 6. Conclusions

Bond properties can be described quantitatively by an atomic dual nature, Ionocovalency (*IC*), which is defined and correlated with quantum-mechanical potential.

Ionocovalency, n*(*I_av_*/*R*)^½^*r_c_*^−1^, which is a dual ionocovalent function of bond strength, charge distribution, charge density, effective ionic potential, or effective polarizing power, is composed of quantum parameters or sub-models, which in turn exhibit versatile specific bond properties and applications, forming a multiple functional *Dual Method*.

The *Dual Method* of multiple-functional prediction of that the dual properties of ionocovalency, which is a bridge of the chemical bond and the potential, should be able to explain fairly well the chemical observations of elements throughout the Periodic Table because they are based on the electron configuration and spectroscopy from 1*s* to n*f.*

Ionocovalency will be further tested against accurate experimental results and in our later papers we shall apply ionocovalency to discuss the types of chemical bonds, the Lewis acid strengths and the glass crosslink density with the Dual Method. And we believe more new applications will be followed by our colleagues.

## Figures and Tables

**Table 1.** Atomic parameters.

**Atm.No**	**Cations**		n*	***I_z_***	***I_av_***	***r_c_^−1^***	n*r_c_^−1^	Z*	***r_c_***	***X_z_***	***X_IC_***	***IC***
1	H^+^		0.85	13.600	13.600	2.703	2.297	0.850	0.370	2.271	**1.333**	**2.297**
2	He^2+^		0.85	54.400	39.500	3.571	3.036	1.449	0.280	6.001	**2.518**	**5.173**
3	Li^+^		1.99	5.3900	5.3900	0.816	1.624	1.253	1.225	0.976	**0.808**	**1.023**
4	Be^2+^		1.99	18.200	13.760	1.031	2.052	2.002	0.970	1.365	**1.237**	**2.064**
5	B^3+^		1.99	37.900	23.800	1.250	2.488	2.633	0.800	2.026	**1.743**	**3.291**
6	C^4+^		1.99	64.500	37.015	1.316	2.618	3.283	0.760	2.583	**2.167**	**4.320**
6	C^3+^		1.99	47.900	27.853	1.316	2.618	2.848	0.760	2.333	**1.931**	**3.747**
6	C^2+^		1.99	24.400	17.830	1.316	2.618	2.279	0.760	1.887	**1.622**	**2.998**
7	N^5+^		1.99	97.900	53.406	1.351	2.689	3.943	0.740	3.125	**2.583**	**5.329**
7	N^4+^		1.99	77.500	42.283	1.351	2.689	3.509	0.740	2.866	**2.341**	**4.742**
7	N^3+^		1.99	47.500	30.543	1.351	2.689	2.982	0.740	2.412	**2.047**	**4.030**
7	N^2+^		1.99	29.600	22.065	1.351	2.689	2.535	0.740	2.067	**1.798**	**3.425**
7	N^+^		1.99	14.530	14.530	1.351	2.689	2.057	0.740	1.680	**1.532**	**2.780**
8	O^6+^		1.99	138.00	72.020	1.370	2.726	4.579	0.730	3.642	**2.972**	**6.273**
8	O^2+^		1.99	35.100	24.360	1.370	2.726	2.663	0.730	2.221	**1.890**	**3.648**
9	F^7+^		1.99	185.00	94.030	1.408	2.803	5.233	0.710	4.284	**3.423**	**7.370**
10	Ne^4+^		1.99	97.100	55.785	1.724	3.431	4.030	0.580	4.584	**3.250**	**6.949**
10	Ne^2+^		1.99	41.000	31.270	1.724	3.431	3.018	0.580	3.250	**2.530**	**5.203**
11	Na^+^		2.89	5.1400	5.1400	0.636	1.838	1.777	1.572	0.948	**0.853**	**1.130**
12	Mg^2+^		2.89	15.000	11.325	0.733	2.119	2.637	1.364	1.168	**1.184**	**1.933**
13	Al^3+^		2.89	28.500	17.763	0.826	2.388	3.303	1.210	1.464	**1.512**	**2.730**
13	Al^+^		2.89	5.9900	5.9900	0.826	2.388	1.918	1.210	1.091	**1.040**	**1.585**
14	Si^4+^		2.89	45.100	25.763	0.847	2.449	3.978	1.180	1.686	**1.776**	**3.371**
14	Si^2+^		2.89	16.300	12.225	0.847	2.449	2.740	1.180	1.323	**1.344**	**2.322**
15	P^5+^		2.89	65.000	35.358	0.935	2.701	4.660	1.070	2.105	**2.181**	**4.355**
15	P^4+^		2.89	51.400	27.948	0.935	2.701	4.143	1.070	1.958	**1.982**	**3.872**
15	P^3+^		2.89	30.200	20.130	0.935	2.701	3.516	1.070	1.682	**1.741**	**3.286**
15	P^+^		2.89	10.490	10.490	0.935	2.701	2.538	1.070	1.309	**1.364**	**2.372**
16	S^6+^		2.89	88.000	46.077	0.971	2.806	5.319	1.030	2.445	**2.515**	**5.165**
16	S^4+^		2.89	47.300	28.940	0.971	2.806	4.216	1.030	1.999	**2.073**	**4.093**
16	S^2+^		2.89	23.300	16.830	0.971	2.806	3.215	1.030	1.634	**1.673**	**3.121**
17	Cl^7+^		2.89	114.00	58.381	1.010	2.919	5.988	0.990	2.832	**2.879**	**6.048**
17	Cl^5+^		2.89	67.800	39.534	1.010	2.919	4.927	0.990	2.362	**2.438**	**4.977**
17	Cl^3+^		2.89	39.600	25.457	1.010	2.919	3.954	0.990	1.988	**2.032**	**3.994**
17	Cl^+^		2.89	12.970	12.970	1.010	2.919	2.822	0.990	1.469	**1.562**	**2.851**
18	Ar^4+^		2.89	59.800	35.965	1.031	2.979	4.700	0.970	2.327	**2.383**	**4.845**
18	Ar^2+^		2.89	27.600	21.680	1.031	2.979	3.649	0.970	1.830	**1.937**	**3.762**
19	K^+^		3.45	4.3400	4.3400	0.513	1.769	1.949	1.950	0.899	**0.799**	**0.999**
20	Ca^2+^		3.45	11.900	9.0050	0.576	1.987	2.807	1.736	1.033	**1.053**	**1.617**
21	Sc^3+^		3.45	24.800	14.713	0.695	2.397	3.588	1.439	1.317	**1.414**	**2.494**
22	Ti^4+^		3.45	43.300	22.805	0.755	2.606	4.468	1.324	1.621	**1.777**	**3.374**
22	Ti^3+^		3.45	27.500	15.973	0.755	2.606	3.739	1.324	1.449	**1.550**	**2.824**
22	Ti^2+^		3.45	13.600	10.210	0.755	2.606	2.989	1.324	1.249	**1.317**	**2.258**
23	V^5+^		3.45	65.200	32.528	0.817	2.819	5.336	1.224	1.990	**2.183**	**4.359**
23	V^4+^		3.45	46.700	24.360	0.817	2.819	4.617	1.224	1.803	**1.941**	**3.772**
23	V^3+^		3.45	29.300	16.913	0.817	2.819	3.847	1.224	1.590	**1.682**	**3.143**
23	V^2+^		3.45	14.700	10.720	0.817	2.819	3.063	1.224	1.352	**1.418**	**2.502**
23	V^+^		3.45	6.7400	6.7400	0.817	2.819	2.429	1.224	1.166	**1.205**	**1.984**
24	Cr^6+^		3.45	90.600	43.878	0.853	2.944	6.197	1.172	2.337	**2.565**	**5.287**
24	Cr^5+^		3.45	69.300	34.534	0.853	2.944	5.498	1.172	2.141	**2.320**	**4.691**
24	Cr^4+^		3.45	49.100	25.843	0.853	2.944	4.756	1.172	1.925	**2.059**	**4.058**
24	Cr^3+^		3.45	31.000	18.090	0.853	2.944	3.979	1.172	1.689	**1.786**	**3.395**
24	Cr^2+^		3.45	16.500	11.635	0.853	2.944	3.191	1.172	1.442	**1.509**	**2.723**
24	Cr^+^		3.45	6.7700	6.7700	0.853	2.944	2.434	1.172	1.202	**1.243**	**2.077**
25	Mn^7+^		3.45	119.00	56.334	0.856	2.954	7.022	1.168	2.578	**2.864**	**6.012**
25	Mn^6+^		3.45	95.000	45.890	0.856	2.954	6.337	1.168	2.386	**2.622**	**5.426**
25	Mn^5+^		3.45	72.400	36.068	0.856	2.954	5.618	1.168	2.181	**2.369**	**4.810**
25	Mn^4+^		3.45	51.200	26.985	0.856	2.954	4.860	1.168	1.958	**2.101**	**4.161**
25	Mn^3+^		3.45	33.700	18.913	0.856	2.954	4.068	1.168	1.734	**1.822**	**3.483**
25	Mn^2+^		3.45	15.600	11.520	0.856	2.954	3.175	1.168	1.428	**1.507**	**2.719**
25	Mn^+^	3.45		7.4400	7.4400	0.856	2.954	2.552	1.168	1.226	**1.287**	**2.185**
26	Fe^6+^	3.45		99.000	47.262	0.858	2.961	6.431	1.165	2.428	**2.661**	**5.520**
26	Fe^5+^	3.45		75.000	36.914	0.858	2.961	5.684	1.165	2.214	**2.397**	**4.879**
26	Fe^4+^	3.45		54.800	27.393	0.858	2.961	4.896	1.165	2.005	**2.119**	**4.203**
26	Fe^3+^	3.45		30.700	18.257	0.858	2.961	3.997	1.165	1.695	**1.801**	**3.431**
26	Fe^2+^	3.45		16.200	12.035	0.858	2.961	3.245	1.165	1.444	**1.535**	**2.786**
26	Fe^+^	3.45		7.8700	7.8700	0.858	2.961	2.624	1.165	1.241	**1.315**	**2.253**
27	Co^4+^	3.45		51.300	27.440	0.870	3.000	4.901	1.150	1.996	**2.143**	**4.261**
27	Co^3+^	3.45		33.500	19.487	0.870	3.000	4.130	1.150	1.762	**1.867**	**3.591**
27	Co^2+^	3.45		17.100	12.480	0.870	3.000	3.305	1.150	1.480	**1.571**	**2.874**
27	Co^+^	3.45		7.8600	7.8600	0.870	3.000	2.623	1.150	1.253	**1.327**	**2.281**
28	Ni^4+^	3.45		54.900	28.985	0.901	3.108	5.037	1.110	2.131	**2.256**	**4.537**
28	Ni^3+^	3.45		35.200	20.347	0.901	3.108	4.220	1.110	1.861	**1.953**	**3.802**
28	Ni^2+^	3.45		18.200	12.920	0.901	3.108	3.363	1.110	1.556	**1.635**	**3.029**
28	Ni^+^	3.45		7.6400	7.6400	0.901	3.108	2.586	1.110	1.281	**1.347**	**2.330**
29	Cu^3+^	3.45		36.800	21.610	0.901	3.108	4.349	1.110	1.885	**2.001**	**3.918**
29	Cu^2+^	3.45		20.300	14.015	0.901	3.108	3.502	1.110	1.599	**1.687**	**3.155**
29	Cu^+^	3.45		7.7300	7.7300	0.901	3.108	2.601	1.110	1.284	**1.352**	**2.343**
30	Zn^2+^	3.45		18.000	13.695	0.801	2.762	3.462	1.249	1.388	**1.529**	**2.772**
30	Zn^+^	3.45		9.3900	9.3900	0.801	2.762	2.867	1.249	1.218	**1.333**	**2.295**
31	Ga^3+^	3.45		30.700	19.067	0.803	2.771	4.085	1.245	1.581	**1.739**	**3.281**
31	Ga^+^	3.45		6.0000	6.0000	0.803	2.771	2.292	1.245	1.131	**1.145**	**1.841**
32	Ge^4+^	3.45		45.700	25.925	0.818	2.821	4.763	1.223	1.794	**1.992**	**3.895**
32	Ge^2+^	3.45		15.900	11.900	0.818	2.821	3.227	1.223	1.376	**1.474**	**2.639**
33	As^5+^	3.45		62.600	33.902	0.826	2.851	5.447	1.210	1.993	**2.242**	**4.502**
33	As^3+^	3.45		28.400	18.937	0.826	2.851	4.071	1.210	1.596	**1.773**	**3.364**
33	As^+^	3.45		9.810	9.8100	0.826	2.851	2.930	1.210	1.257	**1.385**	**2.422**
34	Se^6+^	3.45		81.700	42.442	0.855	2.949	6.095	1.170	2.264	**2.533**	**5.209**
34	Se^4+^	3.45		42.900	26.163	0.855	2.949	4.785	1.170	1.854	**2.072**	**4.090**
34	Se^2+^	3.45		21.200	15.480	0.855	2.949	3.681	1.170	1.533	**1.683**	**3.146**
35	Br^7+^	3.45		103.00	52.601	0.876	3.021	6.785	1.142	2.529	**2.835**	**5.941**
35	Br^5+^	3.45		59.700	35.322	0.876	3.021	5.560	1.142	2.111	**2.393**	**4.869**
35	Br^+^	3.45		11.810	11.810	0.876	3.021	3.215	1.142	1.369	**1.547**	**2.815**
36	Kr^6+^	3.45		78.500	45.200	0.862	2.974	6.290	1.160	2.260	**2.621**	**5.422**
36	Kr^4+^	3.45		52.500	32.000	0.862	2.974	5.292	1.160	1.989	**2.267**	**4.562**
36	Kr^2+^	3.45		24.500	19.250	0.862	2.974	4.105	1.160	1.604	**1.845**	**3.538**
37	Rb^+^	3.85		4.1800	4.1800	0.463	1.782	2.134	2.160	0.885	**0.794**	**0.988**
38	Sr^2+^	3.85		11.000	8.3500	0.522	2.011	3.017	1.914	1.003	**1.036**	**1.576**
39	Y^3+^	3.85		20.500	13.027	0.619	2.382	3.768	1.616	1.211	**1.348**	**2.332**
40	Zr^4+^	3.85		34.300	19.310	0.688	2.648	4.588	1.454	1.472	**1.687**	**3.155**
40	Zr^3+^	3.85		23.000	14.313	0.688	2.648	3.950	1.454	1.346	**1.506**	**2.716**
40	Zr^2+^	3.85		13.100	9.9700	0.688	2.648	3.296	1.454	1.206	**1.321**	**2.267**
41	Nb^5+^	3.85		50.600	27.016	0.745	2.869	5.426	1.342	1.769	**2.053**	**4.043**
41	Nb^4+^	3.85		38.300	21.120	0.745	2.869	4.798	1.342	1.640	**1.860**	**3.575**
41	Nb^3+^	3.85		25.000	15.393	0.745	2.869	4.096	1.342	1.474	**1.644**	**3.052**
41	Nb^2+^	3.85		14.300	10.590	0.745	2.869	3.397	1.342	1.303	**1.430**	**2.532**
41	Nb^+^	3.85		6.8800	6.8800	0.745	2.869	2.738	1.342	1.141	**1.228**	**2.040**
42	Mo^6+^	3.85		68.000	37.683	0.775	2.982	6.409	1.291	2.020	**2.432**	**4.964**
42	Mo^5+^	3.85		61.200	31.620	0.775	2.982	5.870	1.291	1.956	**2.260**	**4.547**
42	Mo^+4^	3.85		46.400	24.225	0.775	2.982	5.138	1.291	1.803	**2.027**	**3.980**
42	Mo^+3^	3.85		27.200	16.833	0.775	2.982	4.283	1.291	1.562	**1.754**	**3.318**
42	Mo^+2^	3.85		16.200	11.650	0.775	2.982	3.563	1.291	1.383	**1.524**	**2.760**
42	Mo^+^	3.85		7.1000	7.1000	0.775	2.982	2.782	1.291	1.177	**1.275**	**2.155**
43	Tc^7+^	3.85		94.000	46.297	0.787	3.031	7.103	1.270	2.287	**2.691**	**5.593**
43	Tc^6+^	3.85		76.000	38.347	0.787	3.031	6.465	1.270	2.135	**2.484**	**5.090**
43	Tc^5+^	3.85		59.000	30.816	0.787	3.031	5.795	1.270	1.973	**2.267**	**4.563**
43	Tc^4+^	3.85		43.000	23.770	0.787	3.031	5.090	1.270	1.798	**2.038**	**4.008**
43	Tc^3+^	3.85		29.500	17.360	0.787	3.031	4.350	1.270	1.622	**1.798**	**3.425**
43	Tc^2+^	3.85		15.300	11.290	0.787	3.031	3.508	1.270	1.385	**1.525**	**2.762**
43	Tc^+^	3.85		7.2800	7.2800	0.787	3.031	2.817	1.270	1.196	**1.301**	**2.218**
44	Ru^8+^	3.85		119.00	57.771	0.806	3.102	7.935	1.241	2.557	**3.021**	**6.394**
44	Ru^7+^	3.85		100.00	49.024	0.806	3.102	7.310	1.241	2.409	**2.814**	**5.890**
44	Ru^6+^	3.85		81.000	40.528	0.806	3.102	6.646	1.241	2.245	**2.593**	**5.355**
44	Ru^5+^	3.85		63.000	32.434	0.806	3.102	5.946	1.241	2.072	**2.361**	**4.791**
44	Ru^4+^	3.85		46.500	24.793	0.806	3.102	5.198	1.241	1.889	**2.113**	**4.189**
44	Ru^3+^	3.85		28.500	17.557	0.806	3.102	4.374	1.241	1.647	**1.839**	**3.525**
44	Ru^2+^	3.85		16.800	12.085	0.806	3.102	3.629	1.241	1.445	**1.592**	**2.924**
45	Rh^6+^	3.85		85.000	42.377	0.802	3.087	6.796	1.247	2.267	**2.632**	**5.450**
45	Rh^4+^	3.85		45.600	25.565	0.802	3.087	5.279	1.247	1.868	**2.131**	**4.233**
45	Rh^3+^	3.85		31.100	18.887	0.802	3.087	4.537	1.247	1.677	**1.886**	**3.638**
45	Rh^2+^	3.85		18.100	12.780	0.802	3.087	3.732	1.247	1.463	**1.620**	**2.993**
45	Rh^+^	3.85		7.4600	7.4600	0.802	3.087	2.851	1.247	1.217	**1.329**	**2.287**
46	Pd^6+^	3.85		90.000	44.273	0.782	3.013	6.946	1.278	2.236	**2.626**	**5.435**
46	Pd^5+^	3.85		66.000	35.128	0.782	3.013	6.188	1.278	2.026	**2.382**	**4.842**
46	Pd^4+^	3.85		49.000	27.410	0.782	3.013	5.466	1.278	1.853	**2.149**	**4.277**
46	Pd^3+^	3.85		32.900	20.210	0.782	3.013	4.693	1.278	1.659	**1.900**	**3.672**
46	Pd^2+^	3.85		19.400	13.870	0.782	3.013	3.888	1.278	1.453	**1.640**	**3.042**
47	Ag^3+^	3.85		34.600	21.227	0.747	2.875	4.810	1.339	1.600	**1.867**	**3.592**
47	Ag^2+^	3.85		21.500	14.540	0.747	2.875	3.981	1.339	1.426	**1.612**	**2.973**
47	Ag^+^	3.85		7.5800	7.5800	0.747	2.875	2.874	1.339	1.161	**1.271**	**2.147**
48	Cd^2+^	3.85		16.900	12.945	0.708	2.725	3.756	1.413	1.293	**1.482**	**2.658**
48	Cd^+^	3.85		8.9900	8.9900	0.694	2.674	3.130	1.440	1.139	**1.283**	**2.174**
49	In^3+^	3.85		28.000	17.563	0.668	2.572	4.375	1.497	1.369	**1.591**	**2.923**
49	In^+^	3.85		5.7900	5.7900	0.668	2.572	2.512	1.497	1.045	**1.078**	**1.678**
50	Sn^4+^	3.85		40.700	23.285	0.715	2.752	5.038	1.399	1.595	**1.871**	**3.601**
50	Sn^2+^	3.85		14.600	10.970	0.715	2.752	3.458	1.399	1.266	**1.405**	**2.472**
51	Sb^5+^	3.85		55.500	30.028	0.709	2.730	5.721	1.410	1.718	**2.059**	**4.057**
51	Sb^3+^	3.85		25.300	16.813	0.709	2.730	4.281	1.410	1.412	**1.638**	**3.036**
52	Te^6+^	3.85		70.700	37.085	0.730	2.810	6.358	1.370	1.902	**2.299**	**4.641**
52	Te^4+^	3.85		37.400	23.253	0.730	2.810	5.034	1.370	1.595	**1.901**	**3.675**
52	Te^2+^	3.85		18.600	13.810	0.730	2.810	3.880	1.370	1.353	**1.554**	**2.832**
53	I^7+^	3.85		92.344	46.535	0.750	2.886	7.122	1.334	2.134	**2.587**	**5.339**
53	I^5+^	3.85		52.395	23.794	0.750	2.886	5.092	1.334	1.798	**1.960**	**3.817**
53	I^+^	3.85		10.450	10.450	0.750	2.886	3.375	1.334	1.232	**1.429**	**2.530**
54	Xe^8+^	3.85		112.91	55.46	0.714	2.750	7.775	1.400	2.139	**2.675**	**5.553**
54	Xe^6+^	3.85		68.718	39.18	0.714	2.750	6.534	1.400	1.839	**2.310**	**4.667**
54	Xe^4+^	3.85		43.956	27.35	0.714	2.750	5.459	1.400	1.626	**1.994**	**3.900**
54	Xe^2+^	3.85		21.200	16.670	0.714	2.750	4.262	1.400	1.366	**1.641**	**3.044**
55	Cs^+^	4.36		3.8900	3.8900	0.426	1.855	2.332	2.350	0.877	**0.796**	**0.992**
56	Ba^2+^	4.36		10.000	7.6050	0.505	2.201	3.260	1.981	1.005	**1.065**	**1.646**
57	La^3+^	4.36		19.200	11.960	0.534	2.330	4.089	1.871	1.132	**1.287**	**2.185**
58	Ce^4+^	4.36		36.720	18.311	0.608	2.649	5.059	1.646	1.412	**1.653**	**3.074**
58	Ce^3+^	4.36		20.199	12.174	0.608	2.649	4.125	1.646	1.248	**1.420**	**2.506**
59	Pr^4+^	4.36		38.979	17.918	0.607	2.646	5.005	1.648	1.430	**1.638**	**3.037**
59	Pr^3+^	4.36		21.619	10.898	0.607	2.646	3.903	1.648	1.263	**1.363**	**2.368**
60	Nd^4+^	4.36		40.420	19.676	0.609	2.655	5.244	1.642	1.447	**1.703**	**3.194**
60	Nd^3+^	4.36		22.075	12.762	0.609	2.655	4.223	1.642	1.272	**1.447**	**2.572**
60	Nd^2+^	4.36		10.716	8.105	0.609	2.655	3.366	1.642	1.121	**1.232**	**2.050**
61	Pm^3+^	4.36		22.283	12.914	0.613	2.675	4.249	1.630	1.281	**1.461**	**2.606**
62	Sm^3+^	4.36		23.423	13.373	0.602	2.627	4.323	1.660	1.275	**1.460**	**2.604**
63	Eu^3+^	4.36		24.874	13.929	0.541	2.357	4.412	1.850	1.190	**1.370**	**2.385**
63	Eu^2+^	4.36		11.245	8.4570	0.541	2.357	3.438	1.850	1.054	**1.153**	**1.858**
64	Gd^3+^	4.36		20.624	12.962	0.620	2.701	4.256	1.614	1.272	**1.474**	**2.637**
64	Gd^2+^	4.36		12.126	9.1310	0.620	2.701	3.572	1.614	1.156	**1.299**	**2.213**
65	Tb^4+^	4.36		39.798	19.759	0.628	2.739	5.255	1.592	1.484	**1.747**	**3.301**
65	Tb^3+^	4.36		21.868	13.079	0.628	2.739	4.276	1.592	1.301	**1.494**	**2.686**
66	Dy^3+^	4.36		22.801	13.466	0.629	2.744	4.339	1.589	1.314	**1.512**	**2.730**
67	Ho^3+^	4.36		22.801	13.542	0.633	2.759	4.351	1.580	1.320	**1.521**	**2.754**
68	Er^3+^	4.36		22.697	13.577	0.638	2.782	4.356	1.567	1.328	**1.532**	**2.780**
69	Tm^3+^	4.36		23.671	13.970	0.640	2.791	4.419	1.562	1.343	**1.553**	**2.829**
69	Tm^2+^	4.36		12.053	9.1190	0.640	2.791	3.570	1.562	1.180	**1.329**	**2.286**
70	Yb^3+^	4.36		25.029	14.487	0.589	2.566	4.500	1.699	1.269	**1.478**	**2.649**
70	Yb^2+^	4.36		12.178	9.2160	0.589	2.566	3.589	1.699	1.119	**1.257**	**2.112**
71	Lu^3+^	4.36		20.956	13.423	0.598	2.609	4.332	1.671	1.242	**1.455**	**2.592**
72	Hf^4+^	4.36		33.300	19.538	0.693	3.024	5.226	1.442	1.566	**1.880**	**3.624**
72	Hf^3+^	4.36		23.300	14.950	0.693	2.024	4.571	1.442	1.436	**1.693**	**3.170**
72	Hf^2+^	4.36		14.900	10.775	0.693	2.024	3.881	1.442	1.304	**1.496**	**2.691**
73	Ta^5+^	4.36		45.000	24.898	0.745	3.246	5.899	1.343	1.835	**2.197**	**4.393**
73	Ta^4+^	4.36		33.100	19.873	0.745	3.246	5.270	1.343	1.684	**2.004**	**3.924**
73	Ta^3+^	4.36		22.300	15.463	0.745	3.246	4.649	1.343	1.521	**1.813**	**3.462**
73	Ta^2+^	4.36		16.200	12.050	0.745	3.246	4.104	1.343	1.411	**1.646**	**3.056**
73	Ta^+^	4.36		7.8900	7.8900	0.745	3.246	3.321	1.343	1.219	**1.406**	**2.473**
74	W^6+^	4.36		61.000	32.363	0.770	3.356	6.726	1.299	2.094	**2.520**	**5.178**
74	W^5+^	4.36		48.000	26.636	0.770	3.356	6.102	1.299	1.945	**2.322**	**4.697**
74	W^4+^	4.36		35.400	21.295	0.770	3.356	5.456	1.299	1.780	**2.117**	**4.200**
74	W^3+^	4.36		24.100	16.593	0.770	3.356	4.816	1.299	1.604	**1.914**	**3.707**
74	W^2+^	4.36		17.700	12.840	0.770	3.356	4.236	1.299	1.485	**1.731**	**3.261**
75	Re^7+^	4.36		79.000	40.311	0.782	3.412	7.506	1.278	2.326	**2.807**	**5.874**
75	Re^6+^	4.36		64.000	33.863	0.782	3.412	6.880	1.278	2.171	**2.605**	**5.383**
75	Re^5+^	4.36		51.000	27.836	0.782	3.412	6.238	1.278	2.021	**2.398**	**4.881**
75	Re^4+^	4.36		37.700	22.045	0.782	3.412	5.551	1.278	1.846	**2.177**	**4.344**
75	Re^3+^	4.36		26.000	16.827	0.782	3.412	4.850	1.278	1.665	**1.950**	**3.795**
75	Re^2+^	4.36		16.600	12.240	0.782	3.412	4.136	1.278	1.486	**1.720**	**3.237**
75	Re^+^	4.36		7.8800	7.8800	0.782	3.412	3.319	1.278	1.265	**1.457**	**2.597**
76	Os^8+^	4.36		99.000	49.325	0.797	3.474	8.303	1.255	2.575	**3.113**	**6.616**
76	Os^7+^	4.36		83.000	42.230	0.797	3.474	7.683	1.255	2.423	**2.909**	**6.122**
76	Os^6+^	4.36		68.000	35.433	0.797	3.474	7.038	1.255	2.267	**2.697**	**5.608**
76	Os^5+^	4.36		54.000	28.920	0.797	3.474	6.358	1.255	2.104	**2.474**	**5.066**
76	Os^4+^	4.36		40.000	22.650	0.797	3.474	5.627	1.255	1.919	**2.234**	**4.483**
76	Os^3+^	4.36		25.000	16.867	0.797	3.474	4.855	1.255	1.680	**1.981**	**3.869**
76	Os^2+^	4.36		16.900	12.800	0.797	3.474	4.230	1.255	1.519	**1.776**	**3.370**
76	Os^+^	4.36		8.7000	8.7000	0.797	3.474	3.487	1.255	1.309	**1.532**	**2.779**
77	Ir^6+^	4.36		72.000	36.683	0.794	3.460	7.161	1.260	2.298	**2.728**	**5.683**
77	Ir^5+^	4.36		57.000	29.620	0.794	3.460	6.434	1.260	2.130	**2.491**	**5.107**
77	Ir^4+^	4.36		39.000	22.775	0.794	3.460	5.642	1.260	1.896	**2.232**	**4.478**
77	Ir^3+^	4.36		27.000	17.367	0.794	3.460	4.927	1.260	1.708	**1.998**	**3.910**
77	Ir^2+^	4.36		16.000	12.550	0.794	3.460	4.188	1.260	1.493	**1.757**	**3.324**
77	Ir^+^	4.36		9.1000	9.1000	0.794	3.460	3.566	1.260	1.316	**1.553**	**2.831**
78	Pt^6+^	4.36		75.000	37.867	0.775	3.380	7.275	1.290	2.258	**2.711**	**5.640**
78	Pt^5+^	4.36		55.000	30.440	0.775	3.380	6.523	1.290	2.045	**2.470**	**5.056**
78	Pt^4+^	4.36		41.100	24.300	0.775	3.380	5.828	1.290	1.873	**2.248**	**4.518**
78	Pt^3+^	4.36		28.500	18.700	0.775	3.380	5.113	1.290	1.689	**2.020**	**3.963**
78	Pt^2+^	4.36		18.600	13.800	0.775	3.380	4.392	1.290	1.513	**1.790**	**3.405**
78	Pt^+^	4.36		9.0000	9.0000	0.775	3.380	3.547	1.290	1.289	**1.520**	**2.749**
79	Au^5+^	4.36		58.000	32.346	0.749	3.263	6.724	1.336	1.991	**2.461**	**5.033**
79	Au^3+^	4.36		30.500	20.077	0.749	3.263	5.297	1.336	1.657	**2.021**	**3.965**
79	Au^2+^	4.36		20.500	14.865	0.749	3.263	4.558	1.336	1.498	**1.793**	**3.412**
79	Au^+^	4.36		9.2300	9.2300	0.749	3.263	3.592	1.336	1.260	**1.495**	**2.689**
80	Hg^2+^	4.33		18.800	14.620	0.694	3.008	4.490	1.440	1.367	**1.672**	**3.118**
80	Hg^+^	4.36		10.440	10.440	0.694	3.028	3.820	1.440	1.219	**1.480**	**2.653**
81	Tl^+3^	4.36		29.800	18.770	0.646	2.815	5.122	1.549	1.423	**1.749**	**3.307**
81	Tl^+^	4.36		6.1100	6.1100	0.646	2.815	2.922	1.549	1.069	**1.164**	**1.887**
82	Pb^4+^	4.36		42.300	24.180	0.650	2.835	5.814	1.538	1.558	**1.944**	**3.780**
82	Pb^2+^	4.36		15.000	11.210	0.650	2.835	3.958	1.538	1.242	**1.447**	**2.574**
83	Bi^5+^	4.36		56.000	30.178	0.658	2.868	6.495	1.520	1.698	**2.147**	**4.273**
83	Bi^+3^	4.36		25.600	16.530	0.658	2.868	4.807	1.520	1.399	**1.690**	**3.162**
84	Po^6+^	4.36		71.488	37.111	0.654	2.850	7.202	1.530	1.804	**2.326**	**4.707**
84	Po^4+^	4.36		37.448	22.922	0.654	2.850	5.660	1.530	0.775	**1.911**	**3.700**
84	Po^3+^	4.36		27.745	18.072	0.654	2.850	5.026	1.530	1.416	**1.740**	**3.285**
84	Po^2+^	4.36		18.051	13.235	0.654	2.850	4.301	1.530	1.292	**1.545**	**2.811**
85	At^7+^	4.36		88.291	44.345	0.690	3.007	7.873	1.450	2.048	**2.624**	**5.430**
85	At^5+^	4.36		88.291	29.370	0.690	3.007	6.407	1.450	2.048	**2.208**	**4.419**
85	At^3+^	4.36		29.262	18.660	0.690	3.007	5.107	1.450	1.508	**1.838**	**3.522**
85	At^+^	4.36		9.2000	9.2000	0.690	3.007	3.586	1.450	1.186	**1.406**	**2.473**
86	Rn^8+^	4.36		106.54	44.700	0.704	3.070	7.904	1.420	2.234	**2.680**	**5.567**
86	Rn^6+^	4.36		65.490	36.730	0.704	3.070	7.165	1.420	1.919	**2.466**	**5.046**
86	Rn^4+^	4.36		41.900	25.250	0.704	3.070	5.941	1.420	1.690	**2.111**	**4.183**
87	Fr^+^	4.36		3.9800	3.9800	0.407	1.772	2.359	2.460	0.869	**0.782**	**0.959**
88	Ra^2+^	4.36		10.200	7.740	0.426	1.855	3.289	2.350	0.940	**0.964**	**1.400**
89	Ac^3+^	4.36		19.692	12.321	0.504	2.199	4.150	1.983	1.097	**1.249**	**2.093**
90	Th^4+^	4.36		28.800	16.595	0.581	2.533	4.816	1.721	1.291	**1.540**	**2.798**
90	Th^3+^	4.36		20.000	12.527	0.581	2.533	4.184	1.721	1.205	**1.389**	**2.431**
91	Pa^4+^	4.36				0.584	2.548	3.900	1.711	1.096	**1.326**	**2.279**
92	U^4+^	4.36				0.594	2.589	3.900	1.684	1.106	**1.341**	**2.316**
93	Np^4+^	4.36				0.600	2.617	3.900	1.666	1.114	**1.351**	**2.341**
94	Pu^4+^	4.36				0.604	2.631	3.900	1.657	1.117	**1.357**	**2.354**
95	Am^4+^	4.36				0.602	2.627	3.900	1.660	1.116	**1.355**	**2.349**
96	Cm^3+^	4.36				0.555	2.421	3.900	1.801	1.065	**1.279**	**2.165**
97	Bk^3+^	4.36				0.568	2.476	3.900	1.761	1.078	**1.299**	**2.215**
98	Cf^3+^	4.36				0.571	2.491	3.900	1.750	1.082	**1.305**	**2.229**
99	Es^3+^	4.36				0.580	2.529	3.900	1.724	1.091	**1.319**	**2.262**
100	Fm^3+^	4.36				0.584	2.547	3.900	1.712	1.096	**1.326**	**2.278**
101	Md^3+^	4.36				0.592	2.581	3.900	1.689	1.104	**1.338**	**2.309**
102	No^3+^	4.36				0.596	2.597	3.900	1.679	1.108	**1.344**	**2.323**
103	Lw^3+^	4.36				1.000	4.360	3.900	1.000	1.715	**1.994**	**3.900**

**Table 2.** *IC* and *X_IC_* for hydrogen and the top elements.

**Elements**	**Li**	**Be**	**H**	**B**	**C**	**N**	**O**	**F**
***IC***	1.023	2.064	2.297	3.291	4.302	5.329	6.273	7.37
***X_IC_***	0.808	1.237	1.333	1.743	2.167	2.583	2.972	3.423

**Table 3.** Point-charge distribution *q_A_* and dipole moment.

**Hydrides**	**LiH**	**BeH**	**BH**	**CH**	**NH**	**OH**	**FH**
**Bond Length (expr)**	1.595	1.343	1.233	1.12	1.038	0.971	0.917
**Dipole Moment (expr)**	5.88	-	-	1.46	-	1.66	1.82
**Dipole Moment (calc)**	5.999	0.281	−1.689	−1.647	−1.743	−1.864	−2.02
***q_A_***	0.783	0.044	−0.285	−0.306	−0.350	−0.400	−0.459

**Table 4.** Bond length and dipole moment

**Bond**	**H-Na**	**H-Mg**	**H-Al**	**H-Si**	**H-Se**	**H-P**	**H-S**	**H-Cl**
**Bond Length (expr)**	1.887	1.73	1.648	1.52	1.475	1.422	1.341	1.275
**Dipole Moment (cal)**	5.966	1.231	−0.169	−0.332	−0.634	−0.651	−1.06	−1.468

**Table 5.** Correlation of *IC* with the standard redox potential *E*^0^ *(M*^2+^/*M)*.

***d^n^***	**0**	**2**	**3**	**4**	**5**	**6**	**7**	**8**	**9**	**10**
***E^0^*/*V***	−2.87	−1.63	−1.18	−0.971	−1.19	−0.44	−0.28	−0.25	0.34	−0.76
***IC***	1.617	2.258	2.502	2.723	2.719	2.786	2.874	3.029	3.155	2.772

**Table 6.** Atomic parameters of Tl, Pb and Bi.

**Cations**	**Tl^+^**	***Tl*^2+^**	**Tl^3+^**	**Pb^2+^**	***Pb^3+^***	**Pb^4+^**	**Bi^3+^**	***Bi^4+^***	**Bi^5+^**
***I_z_***	6.11	**2*0.4***	29.8	15	***3*2**	42.3	25.6	***45.3***	56
***I_av_***	6.11	**1*3.*2*6***	18.77	11.21	**1*8.*1*4***	24.18	16.63	**2*3.7*2**	30.18
***X_IC_***	1.16	**1*.59***	1.75	1.45	**1*.74***	1.94	1.69	**1*.95***	2.15
***IC***	1.89	**2*.9*2**	3.31	2.58	***3.44***	3.78	3.16	***3.8*1**	4.27

**Table 7.** Atomic parameters of Cu, Ag and Au.

**Cations**	**Cu^3+^**	**Ag^3+^**	**Au^3+^**
n*	3.45	3.85	4.36
Z*	4.349	4.81	5.297
***r_c_***	1.11	1.339	1.336
***r_c_*^−1^**	0.901	0.747	0.749
n*r_c_^−1^	3.108	2.875	3.263
***I_av_***	21.610	21.227	18.77
***X_IC_***	1.885	1.867	2.021
***IC***	3.918	3.592	3.965

**Table 8.** *IC*, Z* and δ for Iron-*57*.

**Iron-57**	**Fe^I^**	**Fe^II^**	**Fe^III^**	**Fe^IV^**	**Fe^V^**
**δ/mm s^−1^**	2.3	1.5	0.7	0.2	−0.6
Z* = n*(*I_av_/R*)^½^	2.624	3.245	3.997	4.896	5.684
IC = n*(*I_av_*/*R*)^½^*r_c_*^−1^	2.253	2.786	3.431	4.203	4.879

**Table 9.** Parameters and melting points.

**Compound**	**Cation**	Z*	***I_av_***	***r_c_*^−1^**	n*r_c_^−1^	***X_IC_***	***IC***	**Melt.pt** (**°C**) [43]
KF	K^+^	1.949	4.34	0.513	1.769	0.799	0.999	880⋄
AgF	Ag^+^	2.874	7.58	0.747	2.875	1.271	2.147	435⋄

CaCl_2_	Ca^+2^	2.807	9.005	0.576	1.987	1.053	1.617	772⋄
HgCl_2_	Hg^+2^	4.49	14.82	0.694	3.008	1.672	3.118	276⋄

CaCl_2_	Ca^+2^	2.807	9.005	0.576	1.987	1.053	1.617	772⋄
BeCl_2_	Be^+2^	2.002	13.76	1.125	2.238	1.315	2.252	405⋄

NaBr	Na^+^	1.777	5.14	0.636	1.838	0.853	1.13	755⋄
MgBr_2_	Mg^+2^	2.637	11.33	0.733	2.119	1.184	1.933	700⋄
AlBr_3_	Al^+3^	3.303	17.76	0.826	2.388	1.512	2.73	97.5⋄

KBr	K^+^	1.949	4.34	0.513	1.769	0.799	0.999	730⋄
CaBr_2_	Ca^+2^	2.807	9.005	0.576	1.987	1.053	1.617	765⋄

CsF	Cs^+^	2.332	3.89	0.41	1.787	0.781	0.956	684⋄
BaF_2_	Ba^+2^	3.26	7.605	0.505	2.201	1.065	1.646	1280⋄

**Table 11.** Lattice energies *U* (kJmol^−1^) for MCl_2_ correlate with *IC*.

***d^n^***	**0**	**2**	**3**	**4**	**5**	**6**	**7**	**8**	**9**	**10**
***U***	2260	2500	2580	2580	2550	2650	2700	2790	2840	2760
***IC***	1.617	2.258	2.502	2.723	2.719	2.786	2.874	3.029	3.155	2.772

**Table 12.** Values of log *β* for complexes of 1st row metal ions.

	**Mn^2+^**	**Fe^2+^**	**Co^2+^**	**Ni^2+^**	**Cu^2+^**	**Zn^2+^**
***IC***	2.719	2.786	2.874	3.029	3.155	2.772
***Xic***	1.507	1.535	1.571	1.635	1.687	1.529
**Log *β* for [M(en)_3_]^2−^**	5.7	9.5	13.8	18.6	18.7	12.1
**Log *β* for [M(EDTA)]^2−^**	13.8	14.3	16.3	18.6	18.7	16.1

This order is some time called Irving-Williams series, and is often used in discussing metalloenzyme stabilities (e.g., bioinorganic chemistry).

**Table 13.** Parameters and melting points.

**Compound**	**Cation**	Z*	***I_av_***	***r_c_*^−1^**	n*r_c_^−1^	***X_IC_***	***IC***	**Melt.pt** (**°C**) [43]
KBr	K^+^	1.949	4.34	0.513	1.769	0.799	0.999	730
CaBr_2_	Ca^+2^	2.807	9.005	0.576	1.987	1.053	1.617	765

CsF	Cs^+^	2.332	3.89	0.41	1.787	0.781	0.956	684
BaF_2_	Ba^+2^	3.26	7.605	0.505	2.201	1.065	1.646	1280

**Table 14.** Parameters and lattice energies, −U.

**Compound**	**Cation**	Z*	***I_av_***	***r_c_*^−1^**	n*r_c_^−1^	***X_IC_***	***IC***	***U*** (**kJmol^−1^**) [44]
LiH	Li^+^	1.253	5.39	0.816	2.238	0.808	1.023	905.4
NaH	Na^+^	1.777	5.14	0.636	1.838	0.853	1.13	810.9
KH	K^+^	1.949	4.34	0.513	1.769	0.799	0.999	714.2
